# Single-photon double ionization of ozone: experiment and theory

**DOI:** 10.1038/s41598-026-66986-0

**Published:** 2026-08-22

**Authors:** Veronica Daver Ideböhn, Antoine Gloriod, Richard J. Squibb, Andreas Hult Roos, Nihar Ranjan Behera, Ishita Kanungo, Elias Gustafsson, Simon Gällblad, Saga Berglund, Emelie Olsson, Muneerah Mogren Al-Mogren, Gunnar Öhrwall, Gunnar Nyman, John M. Dyke, John H. D. Eland, Majdi Hochlaf, Raimund Feifel

**Affiliations:** 1 Department of Physics, University of Gothenburg, Origovägen 6B, 412 58 Gothenburg, Sweden; 2 COSYS/IMSE, Université Gustave Eiffel, 5 Bd Descartes, 77454 Champs sur Marne, France; 3 Department of Chemistry, College of Sciences, King Saud University, PO Box 2455, Riyadh, 11451 Saudi Arabia; 4 MAX IV Laboratory, Lund University, Box 118, SE-22100 Lund, Sweden; 5 Department of Chemistry and Molecular Biology, University of Gothenburg, Box 462, 405 30 Gothenburg, Sweden; 6 School of Chemistry and Chemical Engineering, University of Southampton, Highfield, Southampton, SO17 1BJ United Kingdom; 7 Department of Chemistry, Physical and Theoretical Chemistry Laboratory, Oxford University, South Parks Road, Oxford, OX1 3QZ United Kingdom

**Keywords:** Astronomy and planetary science, Chemistry, Physics, Space physics

## Abstract

The triatomic molecule ozone (O$\vphantom{0}_3$) is of outmost physical and chemical importance in the atmospheres of our Earth and other planets. Beyond its environmental significance, a detailed understanding of the electronic structure and ionization dynamics of ozone is essential for modeling atmospheric, ionospheric, and astrochemical processes. In the present work, we substantially extend the experimental and theoretical characterization of ozone into the regime of valence double photoionization. Using He II-*α*, He II-*β*, and higher-energy vacuum ultraviolet radiation in combination with a versatile multiple charged-particle correlation detection technique, we report the first single-photon valence double ionization electron spectrum of O$\vphantom{0}_3$. To interpret the experimental observations, we mapped the lowest potential energy surfaces of O$\vphantom{0}_3^{2+}$ employing post-Hartree-Fock multi-configurational-interaction methods, and computed with high accuracy the energetics of the relevant dissociation channels. Our results demonstrate that dissociative double ionization of ozone produces electronically excited O$\vphantom{0}^+$ and O fragments in addition to the ground-state O$\vphantom{0}_2^+$ + O$\vphantom{0}^+$ dissociation pathway, revealing richer fragmentation dynamics than hitherto recognized.

## Introduction

Ozone (O$\vphantom{0}_3$) is a triatomic molecule of central importance in the chemistry and physics of the Earth and other planetary atmospheres^1^. In the stratosphere, ozone forms a protective layer that absorbs much of the energetic ultraviolet (UV) radiation of the sun, preventing biologically harmful wavelengths from reaching the Earth’s surface and thus protecting living organisms including mankind from DNA and tissue damage. In contrast, high concentrations of ozone in the lower atmosphere (troposphere) constitute a major air pollutant, causing oxidative stress in plants and animals and contributing to respiratory and cardiovascular disease in humans. The dual environmental role of ozone has rendered it a key topic in atmospheric science research for many decades. Following the discovery of the Antarctic ozone hole^2^, intense research efforts have been devoted to understanding both the depletion mechanisms of stratospheric ozone and the formation of tropospheric ozone. These concerns are reflected in international agreements such as the Kyoto^3^ (1997) and Gothenburg protocols^4^ (1999, revised in 2012), which aim to mitigate the adverse effects of ozone imbalance on climate and health.

From a fundamental scientific perspective, detailed knowledge of ozone’s electronic structure, energetics, and reactivity across its various states of charge and excitation is essential for modeling atmospheric and astrochemical processes. The properties of neutral ozone have been extensively investigated using a range of spectroscopic techniques, providing accurate information on its vibrational structure, potential energy surfaces, and symmetry properties. As can be found in chemistry textbooks, the neutral ozone molecule adopts a bent geometry of $\textrm{C}_{2v}$ symmetry, and its ground-state electronic configuration is given by:

$$(1a_1)^2(1b_2)^2(2a_1)^2(3a_1)^2(2b_2)^2(1b_1)^2(4a_1)^2(5a_1)^2(3b_2)^2(1a_2)^2(4b_2)^2(6a_1)^2 \hspace{0.5cm} \mathrm {X^1A_1}$$  

Singly positively charged ozone (O$\vphantom{0}_3^+$) has also been the subject of considerable attention. Its energy levels and vibrational structure have been characterized by vacuum ultraviolet (VUV) photoelectron spectroscopy^5–9^, threshold photoelectron spectroscopy^10^, high-resolution pulsed-field-ionization zero-kinetic-energy (PFI-ZEKE)techniques^11^ and theoretical methods^12,13^. These studies have elucidated the fine structure of the lowest cationic states and revealed strong vibronic coupling and state mixing, reflecting the rich multi-configurational nature of ozone. Core-level photoelectron spectroscopy has further provided site-specific binding energies for the terminal and central oxygen atoms^14^, offering insight into the localization of charge and the dissociation dynamics following core excitation.

By contrast, doubly ionized ozone (O$\vphantom{0}_3^{2+}$) remains much less well understood. From a theoretical standpoint, the removal of two electrons from this highly correlated triatomic system is expected to result in multiple electronic states with complex potential energy surfaces and strong coupling between electronic and nuclear degrees of freedom. Experimentally, only a few studies have addressed the double ionization of ozone so far, most notably those based on electron-impact ionization combined with ion–ion coincidence detection^15^. These experiments identified a threshold of 34.4 eV for forming the lowest-lying dicationic state, which was observed to dissociate into O$\vphantom{0}^+$ and O$\vphantom{0}_2^+$ fragments. Computations suggest that this threshold corresponds to the adiabatic double ionization energy of $\mathrm {O_3}$ (of 34.7–35.0 eV), whereas the vertical double ionization energy is found higher (at *∼*35–36 eV) because of unfavourable Franck-Condon factors upon doubly ionizing ozone (from bent-to-linear structures)^16–18^. Besides, the electron-impact dissociation of $\mathrm {O_3^+}$ is shown to produce $\mathrm {O^+}$ + $\mathrm {O_2^+}$ and $\mathrm {O^+}$ + $\mathrm {O^+}$ + O fragments in addition to $\mathrm {O^+}$ + $\mathrm {O_2}$ and $\mathrm {O^+}$ + O + O channels. The former pathways are most likely due to the unimolecular decomposition of the intermediate $\mathrm {O_3^{2+}}$ dication formed upon ionizing the $\mathrm {O_3^{+}}$cation^19,20^. So far, the $\mathrm {O_3^{2+}}$ parent ion has not been observed, being too short lived to reach the detector. For explanation, Price and co-workers showed that the dicationic potential energy surfaces of singlet – triplet spin cross in the Franck – Condon region upon doubly ionizing $\mathrm {O_3}$. The triplet is dissociative and leads to $\mathrm {O_2^{+}}$ + $\mathrm {O^{+}}$ products and thus prevents measurement of long-lived $\mathrm {O_3^{2+}}$ ions by impact ionization of $\mathrm {O_3}$^16^. While such measurements have provided already some valuable energetic benchmarks, the valence double ionization electron spectrum of ozone has not previously been measured, and little is known about its photon-induced fragmentation dynamics or the stability of its excited dicationic electronic states.

In the present work, we extend substantially the experimental and theoretical characterization of ozone further into the realm of double ionization. Using He II-*α*, He II-*β* and higher energy vacuum ultraviolet radiation and a versatile multiple charged-particle correlation detection method, we have recorded the first single-photon valence double ionization electron spectrum of O$\vphantom{0}_3$. The choice of He II-*β* radiation is particularly relevant, as helium emission is a dominant component of the solar spectrum, rendering these results directly applicable to photoionization and ion–molecule processes in the Earth’s ionosphere and in other planetary atmospheres. The measured double ionization energies of O$\vphantom{0}_3$ provide new benchmarks for theory and enable an improved understanding of the energy available for the dissociation and chemical reactivity of ionic O$\vphantom{0}_3$. For interpretation of the experimental observations, we mapped the lowest potential energy surfaces of ozone dication using post-Hartree-Fock multiconfiguration interaction approaches. We also computed with high accuracy the energetics of $\mathrm {O_3^{2+}}$ and its dissociation channels. With these results, we aim to elevate our fundamental understanding of ozone’s electronic structure well beyond single-ionization and to contribute to a more comprehensive picture of the energy available for ion-molecule reactions of dicationic O$\vphantom{0}_3$ in the Earth’s ionosphere and extraterrestrial planetary atmospheres.

## Experiments

The experiments were carried out in our laboratory at the University of Gothenburg and at the synchrotron radiation facility MAX IV in Lund using time-of-flight photoelectron–photoelectron (TOF-PEPECO) and photoelectron–photoelectron–photoion–photoion–coincidence (TOF-PEPEPIPICO) set-ups based on magnetic bottles, as described previously^21,22^. In Gothenburg, the TOF-PEPECO measurements employed a unique 5.6 m instrument consisting of a conical *∼*1 T magnet in the light–matter interaction region coupled to a solenoid running along the entire flight tube and producing a magnetic field of a few mT. The sample was introduced into the vacuum chamber through a stainless-steel needle, forming an effusive jet in the interaction region. A pulsed helium discharge lamp provided radiation at a repetition rate of about 4.4 kHz, with the photon energy selected using an ellipsoidal grating with a groove density of 1100 lines/mm. This enabled measurements at the discrete photon energies 21.2, 40.8, and 48.4 eV (He I-*α*, He II-*α*, and He II-*β*, respectively) in a small interaction volume. The 5.6 m instrument has a collection–detection efficiency of *∼* 40 % and a nominal resolving power *E/Δ E* of *∼* 120. At the synchrotron radiation facility MAX IV, operated in single bunch mode, the beamline FlexPES^23^ was utilized. This beamline is equipped with a mechanical chopper system to reduce the photon repetiton rate from 3.125 MHz to about 94 kHz to allow sufficient time for all the electrons to reach the detector before the next radiation pulse acts. The photon energy used in the measurements was 56.0 eV and the data were collected with a 2.2 m magnetic bottle with a resolving power of *E/Δ E* *∼* 50, while otherwise being very similar to the 5.6 m instrument.

The TOF-PEPEPIPICO measurements were performed in Gothenburg using the 2.2 m instrument in which the conical magnet was replaced by a hollow ring magnet of reduced field strength. A repeller and pulser plate were installed such that suitable voltages were applied *∼*50 ns after ionization to guide the nascent ions into the 0.12 m ion flight tube in the direction opposite to the electron flight tube, triggered once the electrons had left the interaction region as described in Ref^22^. The potentials guiding the ions were adjusted according to Wiley–McLaren conditions to achieve time focussing for mass resolution and linearity between time deviations and initial ion momentum, allowing the deduction of kinetic energy releases^24^. Replacing the conical magnet with a hollow ring magnet and using a shorter flight tube reduces the nominal resolving power of the electrons to *E/Δ E = 20* and yields a collection–detection efficiency of approximately 20 % for ions. The photon energy was selected using an ellipsoidal grating with a groove density of 550 lines/mm and the repetition rate was about 4.4 kHz. To ensure that electrons and ions recorded within the same time window originate from the same ionization event, the ionization rate is ideally kept below 2 %. However, because of comparatively low vapor pressure of ozone at the working temperature of 195 K and thus relatively high background gas contribution in the apparatus, the count rates were chosen to be slightly higher than ideal, leading to some unwanted coincidences. In both the electron-only and electron–ion configurations, a small voltage (*<1* V) was applied across the interaction region to enable collection of low–kinetic-energy electrons.

Ozone was generated using a commercially available ozoniser from C-Lasky and adsorbed onto non-indicating silica gel beads in a U-tube maintained at 195 K using a dry-ice–isopropanol slush bath. The ozone-loaded U-tube was transferred at 195 K to the spectrometer and kept at the same temperature, desorbing from the beads at an acceptable rate. The gas handling system allows pumping the gas and simultaneously introducing it into the spectrometer, providing an ozone sample with only small amounts of $\mathrm {O_2}$, in addition to a very minute contamination of air^5^. To monitor sample purity throughout the measurements, valence single-ionization spectra were taken periodically in all the acquisitions, and additional ion spectra were collected in the case of TOF-PEPEPIPICO measurements. The main contaminants identified were helium, water, molecular oxygen, and molecular nitrogen, where the ionized oxygen molecule can be distinguished from dissociated ozone by peak width from kinetic energy release.

## Theory

Two types of *ab initio* computations were carried out in order to provide an interpretation of the experimental features observed upon doubly photoionizing $\mathrm {O_3}$. We computed the adiabatic (ADIE) and the vertical (VDIE) double ionization energies of O$\vphantom{0}_3$ and mapped its electronic states lying in the 0–8 eV energy range with respect to the electronic ground dicationic state. We used the approaches described in Ref.^25^ as implemented in the MOLPRO package (version 2024)^26^ where the atoms were described using the basis sets^27–29^ of Dunning and co-workers together with their associated density fitting sets^30^ while using the F12 approaches. In the following we will briefly describe these computations.

The potentials of O$\vphantom{0}_3^{2+}$ were mapped using the standard and explicitly correlated versions of the internally contracted multi-reference configuration interaction (MRCI^31–33^ and MRCI-F12^34–36^) methods on top of state-averaging complete active-space self-consistent field (SA-CASSCF) computations^32,37^. In CASSCF, the active space is composed by considering the orbitals from HOMO-9 up to HOMO+4 (i.e. all the 9 valence orbitals and the 4 next virtual orbitals) as active. All valence electrons were correlated. All electronic states having the same spin-multiplicity were averaged together using the state averaging procedure of MOLPRO. At the MRCI or MRCI-F12 level, the active space was constructed by considering all single and double excitations from the CASSCF wavefunctions, retaining only those having CI coefficients *\ge* 0.05. For each spin multiplicity, we asked for 4 states for each C$\vphantom{0}_s$ symmetry component.

To determine ADIEs and VDIEs of the lowest singlet and triplet states of O$\vphantom{0}_3^{2+}$, we used the (R)CCSD(T)-F12b/aug-cc-pVTZ (opt)//RCCSD(T)/aug-cc-pVTZ + *Δ*CV + *Δ*SR + *Δ*ZPVE (SP) composite scheme, where “opt” and “SP” denote full geometrical optimisations without constraints (in the C$\mathrm {_s}$ point group) and single point computations, respectively. (R)CCSD(T)^38–41^ and (R)CCSD(T)-F12^42–45^ are the standard and the explicitly correlated versions of the coupled clusters method with single, double, and perturbative triple excitations employed. *Δ*CV, *Δ*SR, *Δ*ZPVE correspond to the core-valence, scalar relativistic and zero-point vibrational energy corrections. *Δ*ZPVE was evaluated after a PBE0/aug-cc-pVTZ geometry optimisation followed by anharmonic frequency calculations. ADIEs of the lowest singlet and triplet states of O$\vphantom{0}_3^{2+}$ were evaluated as the energy difference between the energies of the respective dicationic electronic state and the one of the neutral ground state, taken at their respective equilibrium geometries, whereas their VDIEs were determined as the energy difference between the energies of the dicationic electronic state and the one of the neutral ground state at the equilibrium geometry of the neutral species. The expected accuracy of the computed ADIEs is 0.02 eV. Then, the upper dication electronic states were located using their relative energies with respect the lowest O$\vphantom{0}_3^{2+}$ singlet as computed at the MRCI-F12/CASSCF/aug-cc-pVTZ level. These computations were done in the C$\vphantom{0}_{2v}$ point group where we requested 4 states per C$\vphantom{0}_{2v}$ symmetry for a given spin multiplicity. The expected accuracy is 0.2 eV or better.

## Results and discussion

### Electronic structure of $\mathrm {O_3^{2+}}$

**Fig. 1:**
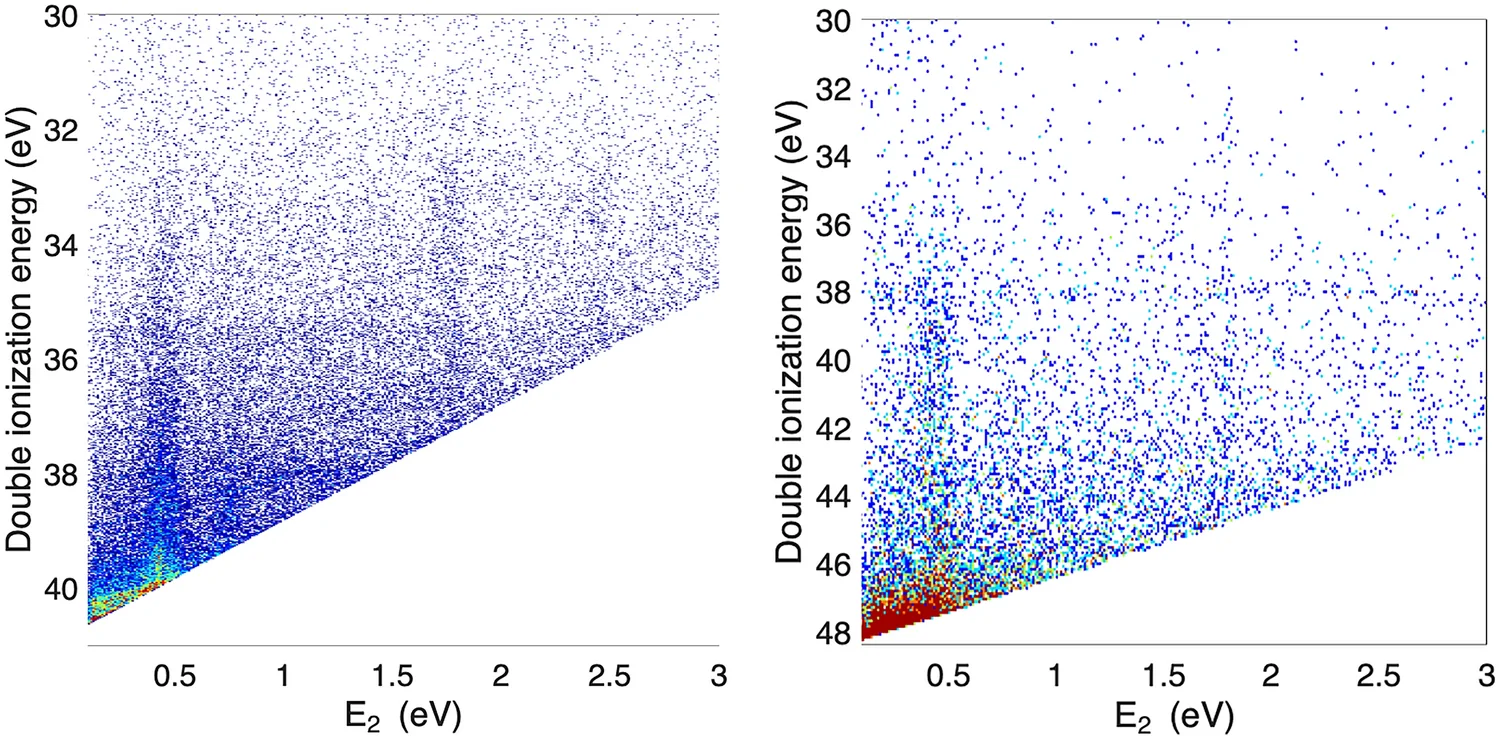
Electron–electron coincidence maps of ozone obtained at 40.8 eV (left) and 48.4 eV (right) photon energies. The most prominent straight vertical lines just below 0.5 eV, around 0.8 eV and close to 1.8 eV agree with energies as previously found in related measurements on molecular oxygen, where they were interpreted as arising from autoionization of atomic oxygen. The horizontal lines are from energy sharing double ionization. In the 40.8 eV data, both the broader band at 35.45 eV and the sharper feature at 38 eV are visible, whereas mainly the 38 eV band is visible in the 48.4 eV data which comprise fewer total counts.

Figure 1 shows electron–electron coincidence maps of ozone obtained at 40.8 and 48.4 eV, with the photon energy minus the electron-pair kinetic energy - i.e., the double-ionization energy - along the vertical axis, and the kinetic energy $E_2$ of the second arrival electron (out of two) along the horizontal axis. A horizontal line at the double-ionization energy of *∼* 38 eV appears as a pronounced feature in these data and reflects a final dicationic electronic state, and in the 40.8 eV data set, a line at 35.5 eV is also visible. In addition, several straight vertical lines are discernible at both photon energies, with the most prominent one just below 0.5 eV and two additonal ones at *∼* 0.8 eV and *∼*1.8 eV. These features match the autoionization features of atomic oxygen, well known from related experiments on molecular oxygen^46^, where their energies were determined as 0.425, 0.495, 0.754, 0.840, 1.685, and 1.785 eV, respectively.

**Fig. 2:**
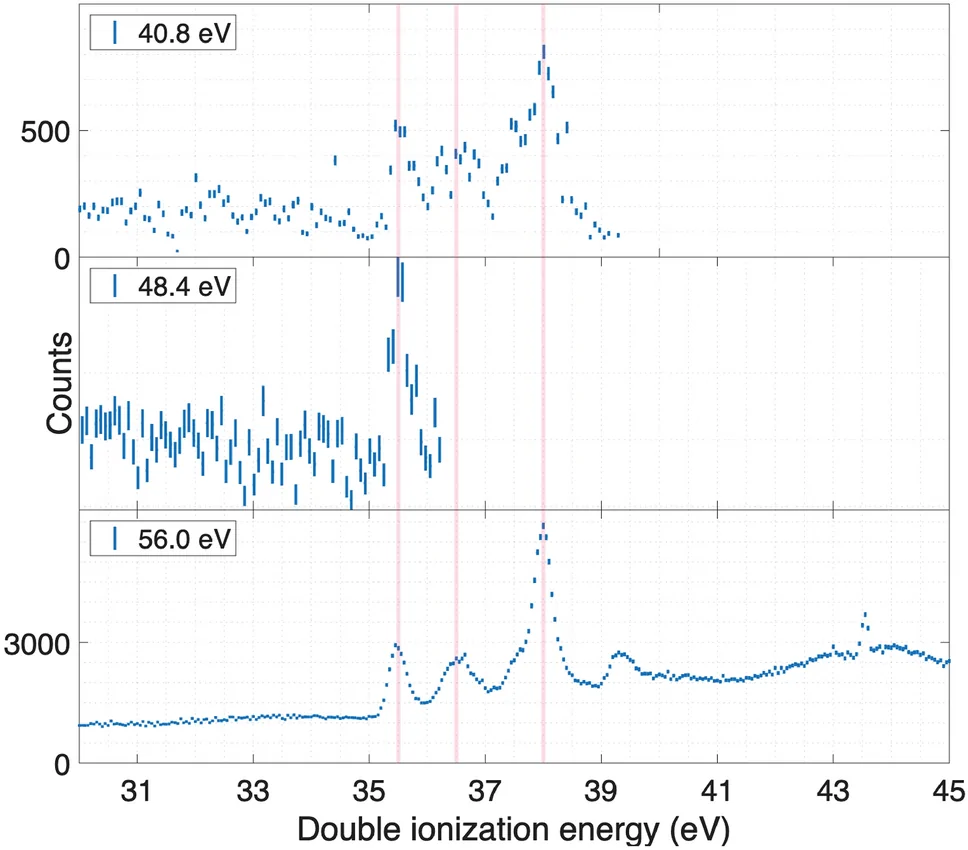
Double ionization electron spectra of ozone taken at 40.8, 48.4 and 56.0 eV photon energy (upper, middle, lower panel respectively). In all spectra, the first, statistically relevant band is located at the vertical ionization energy of 35.5 eV, followed by a broad feature just above 36 eV. The strongest band has a vertical ionization energy of 38 eV. Background has been removed from all spectra to emphasize the features. In the upper two spectra this was done thorough a polynomial subtraction and in the 56.0 eV spectrum, electrons with kinetic energy lower than 3.5 and higher than 52.5 eV have been excluded. Both methods reduce the effect of autoionization electrons and the natural background increases as double ionization energy approaches the photon energy. The sharp band at 43.6 eV in the spectrum taken at 56.0 eV is due to the C $\vphantom{0}^3\Pi _g$ state of O$\vphantom{0}_2^{2+}$ from double ionization of the inevitable presence of O$\vphantom{0}_2$ as a minor impurity^47^. The error bars are statistical and are given by the square root of the number of events, $\mathrm {\sqrt{N}}$. The pink lines are at 35.5, 36.5 and 38 eV and are meant to guide the eye.

Figure 2 shows projections of the coincidence maps from Fig. 1 onto the double-ionization-energy axis, with the addition of a spectrum taken at 56 eV, representing the dicationic electron spectrum of ozone. The 40.8 and 48.4 eV spectra presented have had their background removed, and in the 56.0 eV spectrum, all electrons with kinetic energy lower than 3.5 and higher than 52.5 eV have been removed, in both instances to enhance the spectral features. For the identified bands, the energy resolution is approximately 0.1 eV in the spectrum taken at 48.37 eV and 0.05 eV in the 40.81 eV spectrum. In the 56 eV data, the overall resolving power is just below 0.6 eV for the bands of interest. In these projections, the previously mentioned atomic autoionization lines have been removed from the data. In the 56 eV data, we see traces of $\mathrm {O_2^{2+}}$as a shoulder around 37 eV and as a low intensity peak at 43.6 eV^47^.

**Fig. 3:**
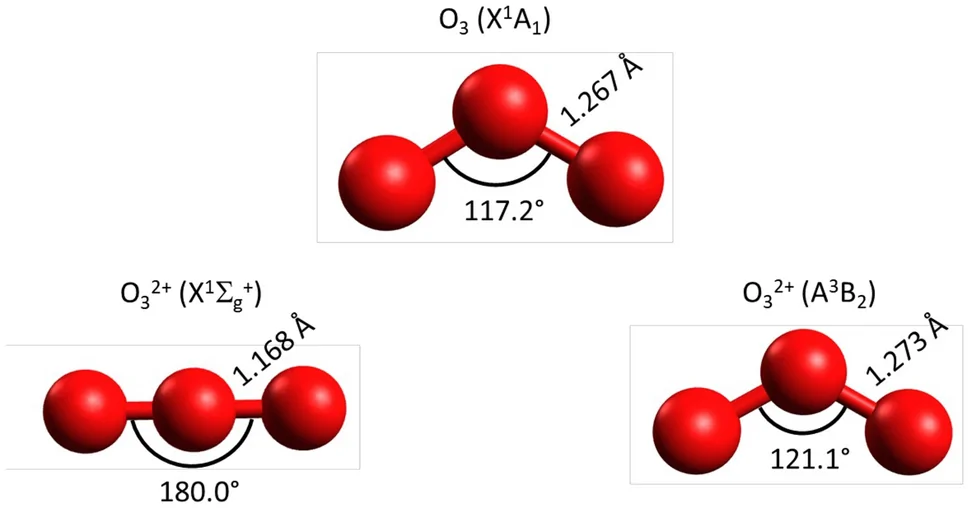
(R)CCSD(T)-F12/aug-cc-pVTZ optimised structures of the ground state of neutral ozone (top) and the two first electronic states of ozone dication (bottom). The geometrical parameters and spectroscopic term are specified.

As seen in Fig. 2, the first band appears at a vertical ionization energy of 35.5 eV, followed by a broader feature around 36.5 eV, and a sharp band at 38 eV. The first band is consistent with the basic “rule of thumb” for double ionization, $\textrm{DIE} = 2.8 \times \textrm{IE}$^48^. Figure 3 shows the optimized structures of the ground state of neutral ozone and those of the lowest singlet and triplet electronic states of ozone dication as computed at the (R)CCSD(T)-F12/aug-cc-pVTZ level. The total energies and geometries in cartesian coordinates at equilibrium are listed in Table S1 in Supplementary materials. The calculations show that the ground electronic state of $\mathrm {O_3^{2+}}$ is a linear singlet of $\mathrm {^1\Sigma _g^+}$ symmetry species, whereas the lowest triplet state is bent and has a $\mathrm {^3B_2}$ spectroscopic term. This is consistent with the earlier theoretical findings of Pyykkö et al.^17,18^ and of Champkin et al.^16^. According to the present calculations, the lowest predicted doubly ionized electronic state is (linear) $\mathrm {X^1\Sigma _g^+}$, see Fig. 3, with an adiabatic double ionisation energy of 34.5 eV and a vertical double ionisation energy of 35.9 eV, both in Table 1. However, calculations show that this state is linear, as seen in Fig. 3, and lies well outside the Franck–Condon region (F.C.), and is therefore expected to appear as a broad, weak feature far from the adiabatic double ionization energy in the double ionization electron spectrum. Consequently, the first experimental band is most likely associated with the lowest triplet state (A$\mathrm {^3B_2}$), for which the adiabatic double ionization energy is computed at 35.5 eV and the vertical double ionization energy is located at 35.6 eV, both listed in Table 1. It is worth noting that we have observed similar shapes for the valence double ionization of SO$\vphantom{0}_2$ populating the $\mathrm {SO_2^{2+}}$electronic states^49^.

**Fig. 4:**
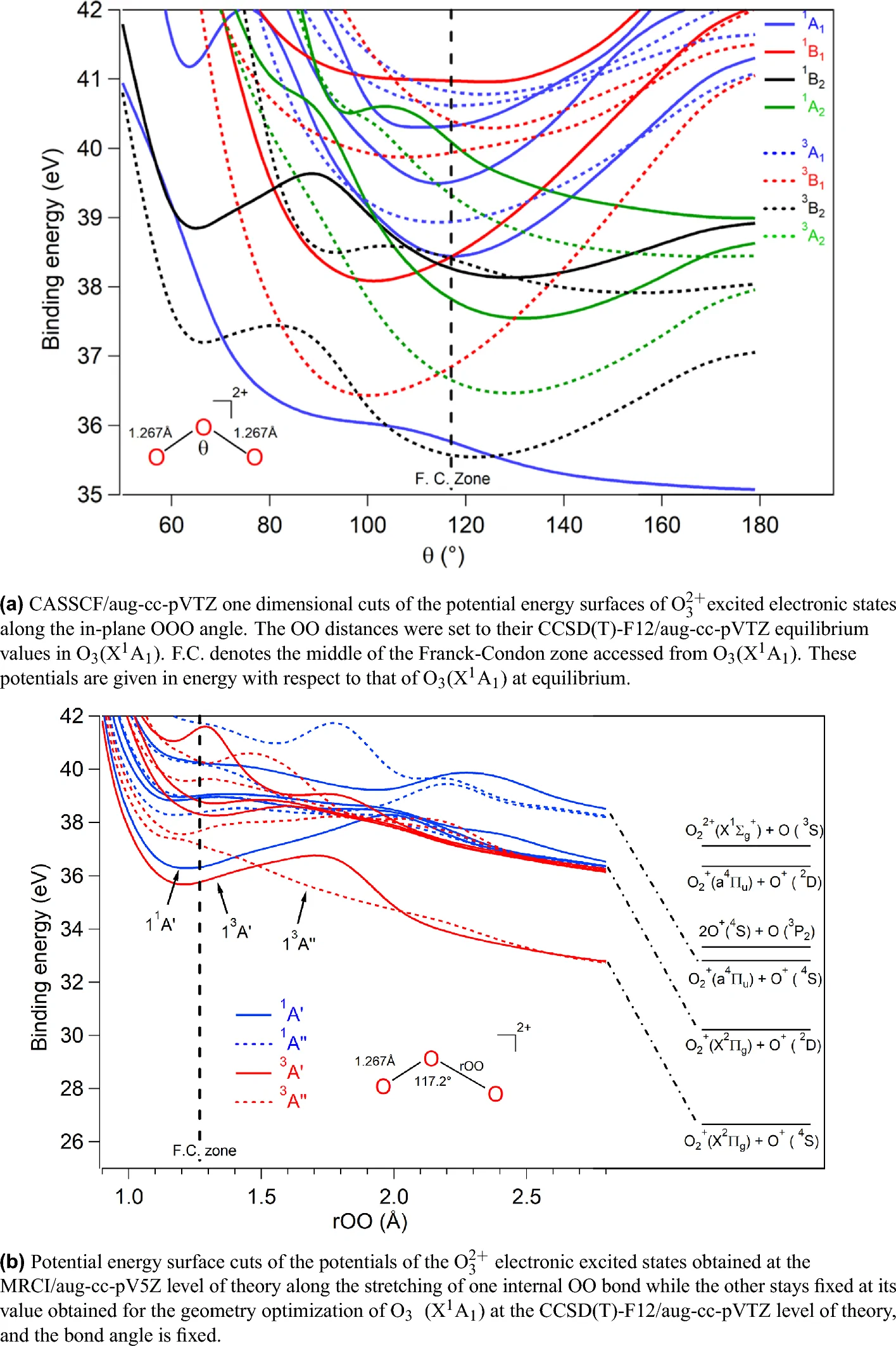
Potential energy surface cuts from altering the angle between terminal oxygen atoms (**a**) and elongating one of the O-O bonds, keeping the other O-O bond and the angle constant (**b**).

Attributing each of the features in the double ionization spectrum to a single electronic state would be an oversimplification. As clearly evidenced by the potential energy surface cuts presented in Figures 4a and 4b, the electronic structure of $\mathrm {O_3^{2+}}$ is characterized by a high density of states, with numerous singlet and triplet states lying within a narrow energy window (36 to 42 eV). These states interact strongly through a variety of non-adiabatic and relativistic spin-orbit induced mechanisms in addition to Renner-Teller effects by bending $\mathrm {O_3^{2+}}$ along the bending coordinate (cf. Fig. 4b). With this in mind, the feature at 35.5 eV can tentatively be assigned as the lowest triplet state, which appears relatively isolated from the other states in the Franck-Condon region at this binding energy. This assignment is supported by two main arguments: the vertical double ionization energy of this state (35.6 eV at MRCI-F12/aug-cc-pVTZ level of theory, see Table 1) is in excellent agreement with the observed band position, and the similar computed geometry of the dication and the ground state of the neutral ozone. This geometric similarity also implies a favourable Franck-Condon overlap, consistent with the relatively sharp appearance of this feature.

In Figure 4a, we give an overview of the potentials of $\mathrm {O_3^{2+}}$along the OOO angle, where both OO distances were kept fixed at their values in $\mathrm {O_3}$($\mathrm {X^1A_1}$). This figure reveals that the electronic structure of $\mathrm {O_3^{2+}}$is characterized by a high density of states, with numerous singlet and triplet states lying within a narrow energy window (35 to 42 eV). Some potentials, such those of the lowest $\vphantom{0}^3$B$\vphantom{0}_1$ and $\vphantom{0}^1$B$\vphantom{0}_1$ electronic states, present two minima for *θ* angles of *∼*120° and *∼*60°. These minima may correpond to stationnay points on such potentials (either stable forms or transition states of $\mathrm {O_3^{2+}}$ dication). This observation has already been made for SO$\vphantom{0}_2$ dication^50^. For confirmation full 3D geometry optimisations followed by frequency computations are needed. The stationnany points far from *θ* *∼*120° are out of the scope of the present work which is mainly concerned by the dicationic states populated in the FC region accessed from the ground state of neutral $\mathrm {O_3}$(i.e. $\theta \,\sim$120°). Moreover, these states interact strongly through a variety of non-adiabatic and spin-orbit interactions. Indeed, Fig. 4a shows that several avoided crossings occur between the electronic states having the same space and spin terms even at low energies. Also, spin-orbit couplings between singlets and triplets at their crossings are possible. In addition, all doubly degenerate electronic states at linearity split into two components upon bending the $\mathrm {O_3^{2+}}$ molecule. Both components are coupled by the Renner-Teller effect. Furthermore, Fig. 4a shows that ground state of $\mathrm {O_3^{2+}}$ has a linear configuration located outside the F.C.zone, whereas the lowest $\mathrm {^3B_2}$ dicationic state is located in this zone. Consequently, unfavourable (favourable) Franck-Condon factors (F.C.Fs) are expected for the $\mathrm {O_3}$($\mathrm {X^1A_1}$) *→* $\mathrm {O_3^{2+}}$($\mathrm {X^1\Sigma _g^+}$) ($\mathrm {O_3}$($\mathrm {X^1A_1}$) *→* $\mathrm {O_3^{2+}}$($\mathrm {A^3B_2}$)) double ionizations. This is in contrast to the isovalent $\mathrm {SO_2^{2+}}$ dication for which the potential of the ground singlet state has no or only weak interactions with the upper states resulting in detectable long-lived $\mathrm {SO_2^{2+}}$ ions in our experiments^50,51^. Thus, different fragmentation mechanisms are expected for $\mathrm {O_3^{2+}}$ compared to $\mathrm {SO_2^{2+}}$.

The optimized structure of $\mathrm {O_3^{2+}}$($\mathrm {X^1\Sigma ^+_g}$) in Fig. 3 shows that the OO distances are shortened by about 0.1 Å upon double ionization of $\mathrm {O_3}$($\mathrm {X^1A_1}$) whereas the OOO angle changes from 117.2° to 180°. This results in unfavourable F.C.Fs upon single photon double ionizing O$\vphantom{0}_3$($\mathrm {X^1A_1}$). Consequently, the 0-0 transition is most unlikely to be observable in the experimental spectra as discussed for the isovalent $\mathrm {SO_2}$/$\mathrm {SO_2^{2+}}$system^49^. In contrast, the first $\mathrm {O_3^{2+}}$triplet (shown in Fig. 3) has a similar equilibrium geometry to the neutral species resulting in favourable F.C.Fs. This is further confirmed upon computing the ADIEs of the $\mathrm {O_3^{2+}}$($\mathrm {X^1\Sigma _g^+, A^3B_2}$) states. The calculated VDIEs and ADIEs are listed in Table 1. At the (R)CCSD(T)-F12b/aug-cc-pVTZ (opt)//(R)CCSD(T)/aug-cc-pVTZ + *Δ*CV + *Δ*SR + *Δ*ZPVE (SP) level, these ADIEs are determined to be 34.4 and 35.5 eV (Table S2). The corresponding VDIEs are 35.9 and 35.6 eV. Table 1 also lists the VDIEs to the upper electronic states of $\mathrm {O_3^{2+}}$, which were obtained by adding the MRCI-F12/aug-cc-pVTZ relative energies to the VDIE of the $\mathrm {O_3^{2+}}$ ($\mathrm {X^1\Sigma _g^+}$) state as evaluated using the (R)CCSD(T)-F12b/aug-cc-pVTZ (opt)//(R)CCSD(T)/aug-cc-pVTZ + *Δ*CV + *Δ*SR (SP) composite scheme. The calculations present a high density of dicationic electronic states in the F.C. region most likely leading to congestion of the corresponding bands in the experimental spectra in Fig. 2.

### Fragmentation dynamics of $\mathrm {O_3^{2+}}$

The mass spectra obtained at a photon energy of 40.8 eV from either single or two ions detected in coincidence with two electrons are presented in the Supplementary Materials, Figure S1. Background signal from air is present in these data as reflected by the features at $\mathrm {m/q} = 18$ ($\mathrm {H_2O}$), 28 ($\mathrm {N_2}$), and 44 ($\mathrm {CO_2}$); the signal at $\mathrm {m/q} = 4$ arises from He leaking in from our gas-discharge lamp. Since no trace of $\mathrm {O_3^{2+}}$ (which should appear at $\mathrm {m/q} = 24$) is observed in either spectrum, these data illustrate that no dicationic electronic state of nascent $\mathrm {O_3^{2+}}$ is sufficiently long-lived to be detected as a stable ion on the time scale of our mass spectrometer. This is in accord with the non-observation of $\mathrm {O_3^{2+}}$formed by electron impact of $\mathrm {O_3}$ in the experiments of Price and co-workers^15^. In addition, calculations are presented in Fig. 4b where one-dimensional cuts of the 3D potential energy surfaces of $\mathrm {O_3^{2+}}$dication along the OO distance are presented, whilst the other OO bond and the OOO angle are kept fixed at 1.267 Å and 117.2° i.e., their values in $\mathrm {O_3}$($\mathrm {X^1A_1}$) at equilibrium. The figure shows a high density of electronic states, leading to their mutual interactions and the mixing of their electronic wavefunctions. For instance, the first $\mathrm {^3A''}$ state is repulsive and crosses the lowest singlet and lowest triplet states not far from the F.C. region, leading to the predissociation. This prevents detecting long-lived $\mathrm {O_3^{2+}}$in the present experiments and those of Newson and Price^52^ as pointed out by Champkin et al.^16^.

**Fig. 5:**
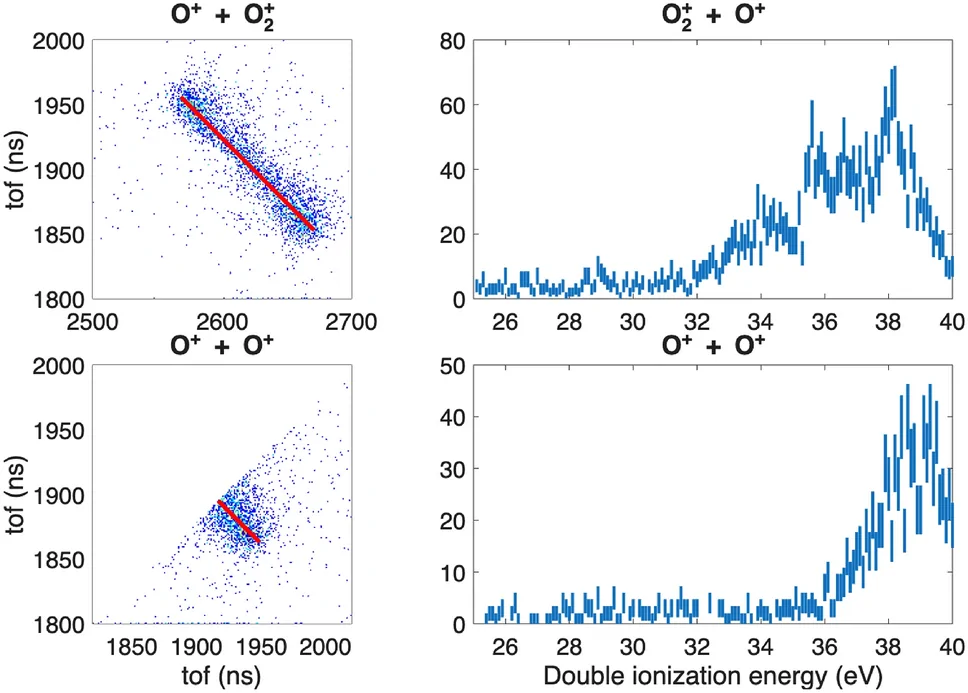
Left side: ion-ion map from electron-electron-ion-ion coincidences, right side: double ionization spectra from electrons in coincidence with ions from the dissociation indicated to the left. The upper map and spectrum corresponds to the $\mathrm {O_3\rightarrow O_2^+ + O^+}$ process. The lower map and spectrum shows the ion island and double ionization energy associated with the $\mathrm {O_3\rightarrow O^+ + O^+}$ process. For the $\mathrm {O^+ + O_2^+}$ coincidences, a structure with bands starting at 35.5 eV and a sharper band near 38 eV are observed. In contrast, for the $\mathrm {O^+ + O^+}$ coincidences, the contribution to the total double-ionization electron signal begins at higher energy, around 36.4 eV, and peaks above 38.5 eV. The structure starting at 32 eV is attributed to auto-ionization and is visibly not from direct double ionization energy sharing. The error bars are statistical and are given by the square root of the number of events, $\mathrm {\sqrt{N}}$.

Figure 5 presents the results of double-ionization measurements on ozone obtained in the electron-ion coincidence mode. The analysis is based on fourfold coincidence events, enabling a direct correlation between the double-ionization energy and specific ion-ion fragmentation channels. The time-of-flight ion-ion coincidence maps from this data selection, which reveal the different dissociation pathways, can also be found to the left of Fig. 5. The upper left panel of Fig. 5 corresponds to $\mathrm {O^+ + O_2^+}$ coincidences, while the lower left panel shows $\mathrm {O^+ + O^+}$ coincidences. In the right spectra in Fig. 5, the central part of the island to the left is left out to exclude any false coincidences from O$\vphantom{0}_2^+$ traces in the sample. This also emphasizes contributions from higher-energy dissociation pathways. The slopes of the red lines in the maps in Fig. 5 are *-1*, indicating that the dissociation predominantly proceeds via a direct breakup channel.

For the $\mathrm {O^+ + O_2^+}$ channel, the spectrum exhibits a structured onset at 35.5 eV, followed by a pronounced band near 38 eV. The signal below 35 eV stems from a two-step process: $\mathrm {O_3 \rightarrow O_3^{+*} \rightarrow O^* + O_2^+: O^* \rightarrow O^+}$, as supported by the electron data where no energy sharing is detected below 35 eV. This process has been identified in measurements on $\mathrm {O_2}$ and is thoroughly explained by Feifel et al.^46^ This is most probably more pronounced here than in the electron only measurements because of the slightly higher pressures used for the electron-ion measurements, as mentioned earlier. In contrast, the $\mathrm {O^+ + O^+}$ channel contributes only at higher energies, with an onset around 36.4 eV and a maximum above 38.5 eV. These results demonstrate that the double-ionization dynamics depend sensitively on the selected ion-ion channel, highlighting the power of multi-particle coincidence measurements for disentangling complex dissociation processes.

Given that the thermodynamic thresholds for the processes in Fig. 5 are significantly lower than the onsets suggest as given in Table 2 the KER becomes interesting to study. For the dissociation limits, the true 0 K values were deduced similarly to Berkowitz et al.^53^ by using the dissociation energy of $\mathrm {O_3 \rightarrow O(^3P_2) + O_2(X ^3\Sigma _g^-)}$ of 1.0621(4) eV^54^ as the starting point for all four processes listed in Table 2. For $\mathrm {O_3 \rightarrow O_2^+ + O^+}$, the first ionization energy of $\mathrm {O_2,\,(O_2^+\,X^2\Pi _g)}$ of 12.07014(15) eV^55^ and the first ionization energy of $\mathrm {O\, ( O^+ \,^4S)}$ of 13.618055(7) eV^56^ are used to produce a 0 K dissociation energy of 26.8 eV. In the case of $\mathrm {O_3 \rightarrow O + 2O^+}$, the first dissociation limit of $\mathrm {O_2 (X^3\Sigma _g^-) \rightarrow O_2^+ (B^2\Sigma _g^-) \rightarrow O(^3P) + O^+(^4S^o)}$ of 18.734 eV^57,58^ replaces the use of the first ionization energy of $\mathrm {O_2}$, leading to a 0 K dissociation limit of 33.4 eV. The energies obtained for the two last processes in Table 2 simply utilize the neutral dissociation energy and the first double ionization energies of the resultants. $\mathrm {O_2}$ has a first double ionization energy of 36.13 ± 0.02 eV^57^ resulting in a 0 K dissociation limit of 37.2 eV for $\mathrm {O_3 \rightarrow O_2^{2+} + O}$ and the second ionization energy of $\textrm{O}$ is reported as 283 270.9 ± 0.5 cm$\vphantom{0}^{-1}$ (35.1211 eV)^59^, giving a 0 K dissociation limit of 49.8 eV for $\mathrm {O_3 \rightarrow O^{2+} + O_2}$. The corresponding computed values at the (R)CCSD(T)-F12/aug-cc-pVTZ level of theory compare favorably to those described in this section. Indeed, the differences are less than 0.1 eV between both data sets.

The spectra in Fig. 5 may be explained by the kinetic energy release (KER) associated with the respective processes, as inferred from the t2-t1 ion time of flight difference peak widths. The KER for the $\mathrm {O_3 \rightarrow O_2^+ + O^+}$ channel has been split up into a lower and higher double ionization energy (DIE) region to evaluate the KER in the first and the last feature of the spectrum in Fig. 5. The lower DIE region, 34–36 eV, is estimated to give a 6.0 eV, and the higher DIE region, 37–40 eV, has an ion peak width correlating to 6.8 eV KER. The KER for the $\mathrm {O_3 \rightarrow 2O^+ + O}$ process has been deduced and is approximately 2.8 eV. The slope of the ion–ion coincidence islands is *-1*(see Supplementary materials), indicating that both processes proceed via two-body dissociation^60^. In the case of the $\mathrm {O_3 \rightarrow 2O^+ + O}$ channel, this implies either that the neutral O atom departs prior to the dissociation of the transient $\mathrm {O_2^{2+}}$ dication, or that the neutral O atom is a pure spectator in immediate dissociation. A possible scenario for the latter case is where the neutral O atom is the central atom of linear $\mathrm {O_3^{2+}}$. The KER values are estimated from the full width at half maximum (FWHM) of the ion–ion coincidence islands, expressed as t2 – t1 (PIPICO peak widths), where the thermal translational velocity contribution cancels. Here, the FWHM of the O_2_ (B $\vphantom{0}^2\Sigma ^-_g$) state following single ionization of O$\vphantom{0}_2$ is used to calculate the field constant, *e*, in the interaction region using its known KER^57^. The FWHM of t$\vphantom{0}_2$-t$\vphantom{0}_1$ of the O$\vphantom{0}^+$+O$\vphantom{0}_2^+$ and O$\vphantom{0}^+$+O$\vphantom{0}^+$ islands in the ion coincidence map following double ionization of ozone are then evaluated. The full FWHM t$\vphantom{0}_2$-t$\vphantom{0}_1$ represents the maximum forward and backward movement following the dissociation, so half the FWHM of the island is used to calculate the kinetic energy of the O$\vphantom{0}^+$ fragment using the field constant derived, *e*. The kinetic energy of O$\vphantom{0}^+$ in either dissociation is equal to half the t$\vphantom{0}_2$-t$\vphantom{0}_1$ FWHM squared multiplied by the field constant, KE(O$\vphantom{0}^+$) = *e*(FWHM/2)$\vphantom{0}^2$. The full interaction also includes the other molecule in the interaction, O$\vphantom{0}^+$ or O$\vphantom{0}_2^+$, which is added to get the full KER. This method of deducing KER is known to risk underestimating the energy released since the high energy wings may be overlooked, so each KER in this article serves as a lower bound. We note that the KER measured in electron impact measurements by Newson and Price^52^are slightly higher than what we have derived from our measurements. They have measured the KER in the same way that we have, but the ionization mechanism is different. We note that the lowest double ionization energy was in the same paper found at 34.3 eV compared to both our first vertical double ionization value at 35.5 eV and appearance energy at about 35.1 eV^52^. 

Using the data in Table 2 together with the potential energy curves of $\mathrm {O_2^+}$^61^ and the known energies of ground and excited states of $\mathrm {O^+}$^56^, some conclusions can be drawn on the final ion states in the dissociation processes that have been identified. Looking at the $\mathrm {O_3 \rightarrow O_2^+ + O^+}$ process, the first part of the spectrum in the 34–36 eV DIE region would leave 2.75 eV available after the dissociation for internal energy, see Table 2. If the process isn’t adiabatic, and the bond length in ozone extends to at least 1.5 Å before the dissociation, the ground state in $\mathrm {O_2^+}$ can hold the excess energy, accounting for the entirety of the internal energy. This is also consistent with the PESs in Fig. 4b where the lowest state, $\mathrm {1^3A'}$ as well as the repulsive state $\mathrm {1 ^3A''}$, dissociates to $\mathrm {O_2^+}$ and $\mathrm {O^+}$ in their ground states with an elongation of the OO bond to 1.8 Å. This elongation would enable up to 4.5 eV as vibrational energy in $\mathrm {O_2^+ (X^2\Pi _g)}$. In addition to the vibrational energy, the $\mathrm {O_2^+}$ ion is likely to hold some rotational energy if $\mathrm {O_3^{2+}}$ is not linear upon dissociation.

Upon formation of $\mathrm {O_3^{2+}}$($\mathrm {A^3B_2}$ (1 $\mathrm {^3A' \ in \ C_s}$ point group)), the reaction may follow the potential of this state and lead to $\mathrm {O_2^+ \;(X^2\Pi _g) + O^+ \; (^2D)}$ after overcoming the potential barrier of the (1 $\mathrm {^1A'}$) state. Also, the $\mathrm {O_3^{2+}}$ $\mathrm {A^3B_2}$ (1 $\mathrm {^1A'}$) may be converted into $\mathrm {O_3^{2+}}$(1 $\mathrm {^3A''}$) at their crossing and then the reaction takes place on the repulsive $\mathrm {O_3^{2+}}$(1 $\mathrm {^3A''}$) potential. Such conversion is allowed by spin-orbit effects (cf. Table S4 in Supplementary materials). This lowers the KER and the AE by few tenths of an eV. The KERs associated with both pathways remain far from those measured (by > 3.5 eV, Table S3). In contrast, close agreement exists between the computed and measured KERs (6.55 or 6.16 eV vs. *∼* 6 eV) if we consider the production of $\mathrm {O_2^+ \;(X^2\Pi _g) + O^+ \; (^2D)}$. Alternatively, the $\mathrm { O_2^+ \;(X^2\Pi _g)}$ ions may be formed in high vibrational levels carrying thus > 3.5 eV internal energy. For illustration, we added in Table 2 the internal energies (E$\mathrm {_{int}}$), depending on the starting $\mathrm {O_3^{2+}}$state formed upon double ionizing $\mathrm {O_3}$. The experimental and the calculated E$\mathrm {_{int}}$ were derived using the following formula: E$\mathrm {_{int}}$ = DIE – E$\mathrm {_{dis}}$ – KER where DIE is the Binding Energy reached upon ionization and E$\mathrm {_{dis}}$ the dissociation energy of each processes.Upon formation of $\mathrm {O_3^{2+}}$($X^1\Sigma _g^+$ (1 $\vphantom{0}^1A'$)), we propose that these ions are converted by spin-orbit effects into the $\mathrm {O_3^{2+}}$ $\mathrm {A^3B_2}$ (1 $\mathrm {^3A'}$)) at the singlet – triplet crossing and then the reaction takes place as just described above. Table S3 shows that such conversion is possible and that a KER of 6.50 eV is associated with such a mechanism. According to the potentials in Fig. 4b, 1 $\mathrm {^3A'}$ exhibits a potential well of 1.1 eV depth, whereas, we compute a much deeper well (of 2 eV) for the 1 $\mathrm {^1A'}$ (= $\mathrm {X^1\Sigma _g^+}$) state, which correlates at large OO distances to the excited $\mathrm {O_2^+ \;(X ^2\Pi _g) + O^+ \; (^2D)}$ asymptote. Assuming that the fragmentation occurs on the singlet (triplet) potential, an AE of 38.3 eV (36.7 eV) and a KER of 8.1 eV (10.1 eV) can be deduced from these potentials (cf. Table S3). Both sets of values are different from those measured previously and in the present work. Clearly, multi-step mechanisms, where several electronic states are involved, appear to be more applicable to these cases.

The higher energy part of the spectrum in Fig. 5, the 37–40 eV DIE region, leaves 4.5 eV as internal energy after the dissociation, see Table 2. Based on the F.C.s of neutral ozone to the ozone dication combined with the potential energy curves of $\mathrm {O_2^+}$ and the energies of $\mathrm {O^+}$, the resultant ions appear to be $\mathrm {O_2^+ \;(X ^2\Pi _g) + O^+ \; (^2D)}$ with 1.2 ($\mathrm {\nu = 5}$) and 3.33 eV internal energy, respectively. This is also reflected in the potential of the $\mathrm {1^1A'}$ dicationic state in Fig. 4b. Based on the potential energy surfaces displayed in Fig. 4b, some dissociations above 37 eV also result in $\mathrm {O_2^+ \;(a^4\Pi _u) + O^+ \; (^4S)}$, where the elongation of the OO bond would allow for an internal energy of up to 2 eV, easily accounting for the excess of 4.5 eV after the dissociation. However, the high density of states above 37 eV makes it complicated to point to a single dominant mechanism in the dissociation. Examination of Table S3 in Supplementary materials reveals that a KER of 6.6 eV (close to the measured 6.8 eV) is expected when the reaction occurs on the $\mathrm {O_3^{2+}}$($3^1A''$) potential leading to the $\mathrm {O_2^+ \;(a^4\Pi _u) + O^+ \; (^4S)}$ excited asymptote. The $\mathrm {O_3^{2+}}$($3^1A''$) state may be populated either directly upon doubly ionizing $\mathrm {O_3}$ or after internal conversion from the upper $\mathrm {O_3^{2+}}$states.

The $\mathrm {O_3^{2+} \rightarrow O^+ + O^+ + O}$ reaction leaves 1.8 eV in internal energy after the dissociation. Since none of the states agree with this kind of internal energy, the remaining O atom is assumed to have kept some of the energy in the dissociation, leaving both the ions in the ground state, $\mathrm {O^+\, (^4S)}$.

**Fig. 6:**
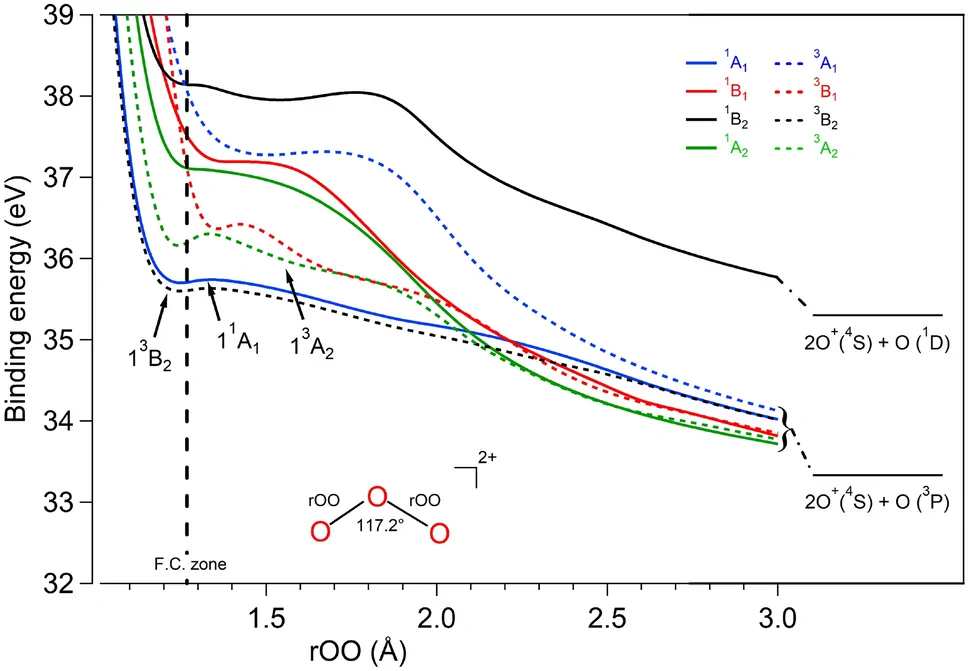
CASSCF/aug-cc-pVTZ one-dimensional cuts of the potential energy surfaces of the lowest electronic states of $\mathrm {O_3^{2+}}$while lengthening simultaneously both OO bonds. The OOO angle is kept fixed at its equilibrium value in $\mathrm {O_3}$($X^1A_1$). The reference energy is that of $\mathrm {O_3}$($\mathrm {X^1A_1}$) at equilibrium.

Figure 6 shows the one-dimensional evolution of the singlet and triplet states of the $\mathrm {O_3}$ dication while lengthening both OO distances symmetrically. These potentials correlate at large interatomic distances to the $\mathrm {O^+ + O^+ + O}$ asymptote. This figure shows that upon population of the dicationic potential wells in the F.C. zone, only small barriers separate them from the 1/r Coulombic evolution before reaching the respective dissociation limit. In particular, the $\mathrm {O_3^{2+}}$electronic states lying in the 35.5–38 eV range lead to the $\mathrm {O^+ + O^+ + O}$ fragments in their electronic ground states, whereas the lowest $\mathrm {^1B_2}$ state leads adiabatically to the $\mathrm {O^+ + O^+ + O(^1D)}$ excited channel. Table S3 in Supplementary materials lists the calculated KERs and AEs deduced from these potentials for the $\mathrm {X^1\Sigma _g^+, A^3B_2 \; and\; 3^1A''}$ states i.e., KER = 2.41, 2.30 and 2.97 eV, respectively, in reasonable agreement with the 2.8 eV determined in the present experiments for the ground state channel.

**Table 1:** Vertical double ionization energies (VDIEs) from the 40.8 eV experimental spectrum compared to calculated adiabatic double ionization energies (ADIEs) and VDIEs to $\mathrm {O_3^{2+}}$electronic states within the 35 to 41 eV range of binding energy. We also give the dominant electronic configuration (in C$\mathrm {_{2v}}$ point group) and the corresponding CI coefficient of these states as quoted at the equilibrium geometry of O_3_. These molecular orbitals are visualized in Figure S2.

State	Dominant electronic configuration	Experiment$\vphantom{0}^{\text {a)}}$	Theory$\vphantom{0}^{\text {a)}}$		Ref^52^	Ref^17^
		VDIE (eV)	ADIE (eV)$\vphantom{0}^{\text {b})}$	VDIE (eV)$\vphantom{0}^\text {c)}$	ADIE (eV)	ADIE (eV)
$\mathrm {X^1\Sigma _g^+}$	0.64 : (core)$\mathrm { (3a_1)^2 (2b_2)^2 (1b_1)^2 (4a_1)^2 (5a_1)^2 }$ $\mathrm {(3b_2)^2 (1a_2)^2 (4b_2)^2 (6a_1)^0 }$		34.445	35.893	34.3 ± 0.3	
$\mathrm {A^3B_2}$	0.86 : (core)$\mathrm { (3a_1)^2 (2b_2)^2 (1b_1)^2 (4a_1)^2 (5a_1)^2 }$ $\mathrm {(3b_2)^2 (1a_2)^2 (4b_2)^1 (6a_1)^1 }$	35.5 ± 0.4	35.513	35.603		35.17$\vphantom{0}^\text {d)}$
						30.70$\vphantom{0}^\text {e)}$
						35.6$\vphantom{0}^\text {f)}$
						35.8$\vphantom{0}^\text {g)}$
$\mathrm {1^3A_2}$	0.87 : (core)$\mathrm { (3a_1)^2 (2b_2)^2 (1b_1)^2 (4a_1)^2 (5a_1)^2 }$ $\mathrm {(3b_2)^2 (1a_2)^1 (4b_2)^2 (6a_1)^1 }$	36.4 ± 0.10		36.713		
$\mathrm {1^3B_1 }$	0.86 : (core)$\mathrm { (3a_1)^2 (2b_2)^2 (1b_1)^2 (4a_1)^2 (5a_1)^2 }$ $\mathrm {(3b_2)^2 (1a_2)^1 (4b_2)^1 (6a_1)^2 }$			36.943		
$\mathrm {1^1A_2 }$	0.88 : (core)$\mathrm { (3a_1)^2 (2b_2)^2 (1b_1)^2 (4a_1)^2 (5a_1)^2 }$ $\mathrm {(3b_2)^2 (1a_2)^1 (4b_2)^2 (6a_1)^1 }$			37.826		
$\mathrm {2^3B_2 }$	0.74 : (core)$\mathrm { (3a_1)^2 (2b_2)^2 (1b_1)^2 (4a_1)^2 (5a_1)^2 }$ $\mathrm {(3b_2)^2 (1a_2)^1 (4b_2)^2 (6a_1)^0 (7a_1)^0 (5b_2)^0 (2b_1)^1}$	38 ± 0.02		38.286		
$\mathrm {1^1B_2 }$	0.77 : (core)$\mathrm { (3a_1)^2 (2b_2)^2 (1b_1)^2 (4a_1)^2 (5a_1)^2 }$ $\mathrm {(3b_2)^2 (1a_2)^2 (4b_2)^1 (6a_1)^1 }$			38.429		
$\mathrm {2^1A_1 }$	0.50 : (core)$\mathrm { (3a_1)^2 (2b_2)^2 (1b_1)^2 (4a_1)^2 (5a_1)^2 }$ $\mathrm {(3b_2)^2 (1a_2)^2 (4b_2)^0 (6a_1)^2 }$			38.449		
$\mathrm {1^1B_1}$	0.87 : (core)$\mathrm { (3a_1)^2 (2b_2)^2 (1b_1)^2 (4a_1)^2 (5a_1)^2 }$ $\mathrm {(3b_2)^2 (1a_2)^1 (4b_2)^1 (6a_1)^2 }$			38.465		
$\mathrm {1^3A_1 }$	0.69 : (core)$\mathrm { (3a_1)^2 (2b_2)^2 (1b_1)^2 (4a_1)^2 (5a_1)^2 }$ $\mathrm {(3b_2)^2 (1a_2)^1 (4b_2)^1 (6a_1)^1 (7a_1)^0 (5b_2)^0 (2b_1)^1}$			38.674		
$\mathrm {2^3A_2 }$	0.87 : (core)$\mathrm { (3a_1)^2 (2b_2)^2 (1b_1)^2 (4a_1)^2 (5a_1)^2 }$ $\mathrm {(3b_2)^2 (1a_2)^2 (4b_2)^1 (6a_1)^0 (7a_1)^0 (5b_2)^0 (2b_1)^1}$			39.113		
$\mathrm {3^1A_1}$	0.82 : (core)$\mathrm { (3a_1)^2 (2b_2)^2 (1b_1)^2 (4a_1)^2 (5a_1)^2 }$ $\mathrm {(3b_2)^2 (1a_2)^0 (4b_2)^2 (6a_1)^2 }$			39.766		
$\mathrm {2^1A_2 }$	0.86 : (core)$\mathrm { (3a_1)^2 (2b_2)^2 (1b_1)^2 (4a_1)^2 (5a_1)^2 }$ $\mathrm {(3b_2)^2 (1a_2)^2 (4b_2)^1 (6a_1)^0 (7a_1)^0 (5b_2)^0 (2b_1)^1}$			39.774		
$\mathrm {2^3B_1 }$	0.79 : (core)$\mathrm { (3a_1)^2 (2b_2)^2 (1b_1)^2 (4a_1)^2 (5a_1)^2 }$ $\mathrm {(3b_2)^2 (1a_2)^2 (4b_2)^2 (6a_1)^1 (7a_1)^0 (5b_2)^0 (2b_1)^1}$			39.914		
$\mathrm {4^1A_1 }$	0.74 : (core)$\mathrm { (3a_1)^2 (2b_2)^2 (1b_1)^2 (4a_1)^2 (5a_1)^2 }$ $\mathrm {(3b_2)^2 (1a_2)^1 (4b_2)^1 (6a_1)^1 (7a_1)^0 (5b_2)^0 (2b_1)^1}$			40.047		
$\mathrm {2^3A_1 }$	0.67 : (core)$\mathrm { (3a_1)^2 (2b_2)^2 (1b_1)^2 (4a_1)^2 (5a_1)^2 }$ $\mathrm {(3b_2)^2 (1a_2)^1 (4b_2)^1 (6a_1)^1 (7a_1)^0 (5b_2)^0 (2b_1)^1}$			40.421		
$\mathrm {3^3B_1 }$	0.63 : (core)$\mathrm { (3a_1)^2 (2b_2)^2 (1b_1)^2 (4a_1)^2 (5a_1)^2 }$ $\mathrm {(3b_2)^2 (1a_2)^0 (4b_2)^2 (6a_1)^1 (7a_1)^0 (5b_2)^0 (2b_1)^1}$			40.570		
$\mathrm {3^3A_1 }$	0.54 : (core)$\mathrm { (3a_1)^2 (2b_2)^2 (1b_1)^2 (4a_1)^2 (5a_1)^2 }$ $\mathrm {(3b_2)^2 (1a_2)^1 (4b_2)^1 (6a_1)^1 (7a_1)^0 (5b_2)^0 (2b_1)^1}$			40.767		
$\mathrm {2^1B_1 }$	0.58 : (core)$\mathrm { (3a_1)^2 (2b_2)^2 (1b_1)^2 (4a_1)^2 (5a_1)^2 }$ $\mathrm {(3b_2)^2 (1a_2)^2 (4b_2)^2 (6a_1)^1 (7a_1)^0 (5b_2)^0 (2b_1)^1}$			40.918		

a) This work;.

b) (R)CCSD(T)-F12/aug-cc-pVTZ (opt)//(R)CCSD(T)/aug-cc-pVTZ +*Δ*CV +*Δ*SR +*Δ*ZPVE (SP) composite scheme;.

c) MRCI-F12/aug-cc-pVTZ calculations with (R)CCSD(T)-F12/aug-cc-pVTZ (opt)//(R)CCSD(T)/aug-cc-pVTZ +*Δ*CV +*Δ*SR composite scheme used as reference for VDIE of$\mathrm {A^3B_2}$state;.

d) HF/6-31G;.

e) MP2/6-31G;.

f) MP3/6-31G;.

g) CISD/6-31G.

**Table 2:** Comparison of the calculated thermodynamic thresholds (True 0 K dissociation energies) for the processes identified in the experimental data and their corresponding associated kinetic energy releases (KER) as derived from the peak widths in the ion-ion coincidence maps. The first reaction has been split into two different DIE regions, 34–36 eV (*) and 37–40 eV (**), as indicated by the asterisks. The True 0 K dissociation energies shown make use of identified appearance energies noted in the literature together with the dissociation energy associated with the $\mathrm {O_3 \rightarrow O + O_2}$process^53–57,59,62^. The calculated corresponding data are found through our (R)CCSD(T)-F12/aug-cc-pVTZ calculations where the identified asymptotes have been listed. The third column lists the KER, derived from the peak widths in the ion-ion coincidence maps. See Table S3 for more details on the theoretical data. In the table, $\mathrm {E_{dis}}$ is the dissociative energy and $\mathrm {E_{int}}$ is the internal energy.

Process	True 0 K E$$\vphantom{0}_\textrm{dis}$$(eV) $$\vphantom{0}^\text {a)}$$	Calc. E$$\mathrm {_{dis}}$$ (eV) $$\vphantom{0}^\text {b)}$$	KER$$\vphantom{0}^\text {c)}$$ (eV)	Lit. $$\vphantom{0}^\text {d)}$$ KER (eV)	$$\mathbf {\mathbf {E_{int}}}$$$$\vphantom{0}^\text {b)}$$ (eV)	Starting $$\mathrm {O_3^{2+}}$$ on DIE	Calc. AE (eV)	Calc. KER (eV)	Calc. $$\mathbf {E_{int}}$$ (eV)
$\mathrm {O_3 \rightarrow O_2^+ + O^+}$(*)	26.75	26.66	6.0	7.5±0.3 7.5±0.3 8.1±0.4	2.75$\vphantom{0}^\text {e)}$	$\mathrm {A^3B_2}$ $\mathrm {A^3B_2-X^1\Sigma ^+_g}$ $\mathrm {A^3B_2-1^3A'}$	36.76 36.71 36.40	6.55 6.50 6.16	3.55 3.55 3.58
$\mathrm {O_3 \rightarrow O_2^+ + O^+}$(**)	26.75	26.66	6.8		4.45$\vphantom{0}^\text {f)}$	$\mathrm {X^1\Sigma ^+_g}$ $\mathrm {3^1A''}$	38.28 39.44	8.08 6.63	3.54 6.15
$\mathrm {O_3 \rightarrow 2O^+ + O}$	33.41	33.33	2.8		1.79$\vphantom{0}^\text {g)}$	$\mathrm {X^1\Sigma ^+_g}$ $\mathrm {A^3B_2}$ $\mathrm {3^1A''}$	35.74 35.63 36.30	2.41 2.30 2.97	0 0 0
$\mathrm {O_3 \rightarrow O_2^{2+} + O}$	37.19	37.12	-						
$\mathrm {O_3 \rightarrow O^{2+} + O_2}$	49.80	49.72	-						

a) Based on values found in^53,55–57,59,62^.

b) Calculations from this work.

c) Experimental data from this work.

d) Electron impact at 55, 150 and 233 eV of electron energy^16^.

e) Using DIE = 35.5 eV.

f) Using DIE = 38 eV.

g) Using DIE = 38 eV.

## Conclusions

In this work, we have substantially advanced the experimental and theoretical understanding of ozone in the regime of valence double photoionization. By combining He II-*α*, He II-*β*, and higher-energy vacuum ultraviolet radiation with a versatile multiple charged-particle correlation detection technique, we recorded the first single-photon valence double-ionization electron spectrum of $\mathrm {O_3}$ and obtained detailed insight into the fragmentation dynamics of the resulting dicationic states. The measured double-ionization energies provide valuable experimental benchmarks for future theoretical descriptions of multiply ionized ozone.

To interpret the experimental observations, we mapped the lowest potential energy surfaces of $\mathrm {O_3^{2+}}$using state-of-the-art post-Hartree–Fock multiconfigurational methods and determined with high accuracy the energetics of the relevant dissociation channels. The combined experimental and theoretical results reveal that dissociative double ionization of ozone proceeds not only through the formation of ground-state O$\vphantom{0}_2^+$ + O$\vphantom{0}^+$ fragments, but also through pathways involving electronically excited O$\vphantom{0}^+$ ions or O atoms. The energetics of the first part of the double ionization spectrum of the $\mathrm {O_3\rightarrow O_2^++O^+}$ combined with the found KER reveals that both O$\vphantom{0}_2^+$ and O$\vphantom{0}^+$ fragments dissociate in the ground electronic state. The energetics of the 38 eV band of the same process suggests that the outgoing O$\vphantom{0}^+$ is electronically excited to $\vphantom{0}^2$D and that the O$\vphantom{0}_2^+$ fragment has substantial vibrational energy of about 1 eV. The potential energy surfaces presented also show that some dissociations above 37 eV double ionization energy result in an electronically excited O$\vphantom{0}_2^+$ (a$\vphantom{0}^4\Pi _u$) and the ground state O$\vphantom{0}^+$ ($\vphantom{0}^4$S). These findings uncover a level of complexity in the fragmentation dynamics of doubly ionized ozone that has not previously been recognized.

Beyond providing a detailed picture of ozone dication decay, the present results offer new insight into the redistribution of energy following molecular double ionization, a process of fundamental importance in radiation-driven environments. The observation of electronically excited oxygen fragments is particularly significant, as such species can initiate highly reactive chemical pathways in planetary atmospheres. In this respect, the processes identified here may represent an ionization-driven analogue to the well-known production of O($\vphantom{0}^1$D) atoms in ozone photodissociation, highlighting a potentially overlooked source of chemical activation under intense ionizing radiation.

Overall, this study establishes the first comprehensive framework for understanding valence double ionization and subsequent fragmentation in ozone. By combining high-resolution experiment and advanced electronic-structure theory, it not only provides definitive benchmarks for future investigations of ozone dications, but also opens new avenues for exploring the role of multiply ionized molecules in atmospheric, planetary, and astrochemical environments.

## Supplementary Information

Supplementary Information 1.

Supplementary Information 2.

## Data Availability

Data is available upon request.
