# Supplementary material for: Single-photon double ionization of ozone: experiment and theory
Source: Sci Rep. 2026 Aug 22;16:26361. doi: 10.1038/s41598-026-66986-0 (PMC13499803; doi:10.1038/s41598-026-66986-0)
Supplement: Supplementary file 2 [file 41598_2026_66986_MOESM2_ESM.pdf]

Supplementary Materials
Single-Photon Double Ionization of Ozone: Experiment and Theory

Veronica Daver Ideböhn[1], Antoine Gloriod[2], Richard J. Squibb[1], Andreas Hult Roos[1], Nihar Ranjan Behera[1], Ishita Kanungo[1], Elias Gustafsson[1], Simon Gällblad[1], Saga Berglund[1], Emelie Olsson[1], Muneerah Mogren Al-Mogren[7], Gunnar Öhrwall[3], Gunnar Nyman[4], John M. Dyke[5], John H.D. Eland[6], Majdi Hochlaf[2,*], and Raimund Feifel[1,]**

[1]University of Gothenburg, Department of Physics, Origovägen 6B, 412 58 Gothenburg, Sweden
[2]Université Gustave Eiffel, COSYS/IMSE, 5 Bd Descartes, 77454 Champs sur Marne, France
[4]MAX IV Laboratory, Lund University, Box 118, SE-22100 Lund, Sweden
[5]University of Gothenburg, Department of Chemistry and Molecular Biology, Box 462, 405 30 Gothenburg, Sweden
[6]School of Chemistry and Chemical Engineering, University of Southampton, Highfield, Southampton SO17 1BJ, United Kingdom
[7]Oxford University, Department of Chemistry, Physical and Theoretical Chemistry Laboratory, South Parks Road, Oxford OX1 3QZ, United Kingdom
[3]Department of Chemistry, College of Sciences, King Saud University, PO Box 2455, Riyadh 11451, Saudi Arabia.
[*]majdi.hochlaf@univ-eiffel.fr
[**]raimund.feifel@physic.gu.se

Figure S1 shows the mass spectrum of triple (blue) and four-fold (pink) electron-ion coincidences from 40.8 eV measurements. Mass/charge (m/q) of 16, 32 and 48 come from the sample O^+, O_2^+ and O_3^+. No trace of the doubly ionized parent, O_3^{2+}, is present which would have presented as a peak at m/q = 24. Traces of O_2 is identified by looking at the width of the m/q = 32 peak where the narrow part of the peak comes from non-dissociated O_2^+ and the broadening of the peak appears as a result of dissociation of O_3^{2+}. We find traces of Helium at m/q = 4, originating from our helium discharge lamp. Moreover, traces of air (water and molecular nitrogen at m/q = 16-18 and 28, respectively) are also present.

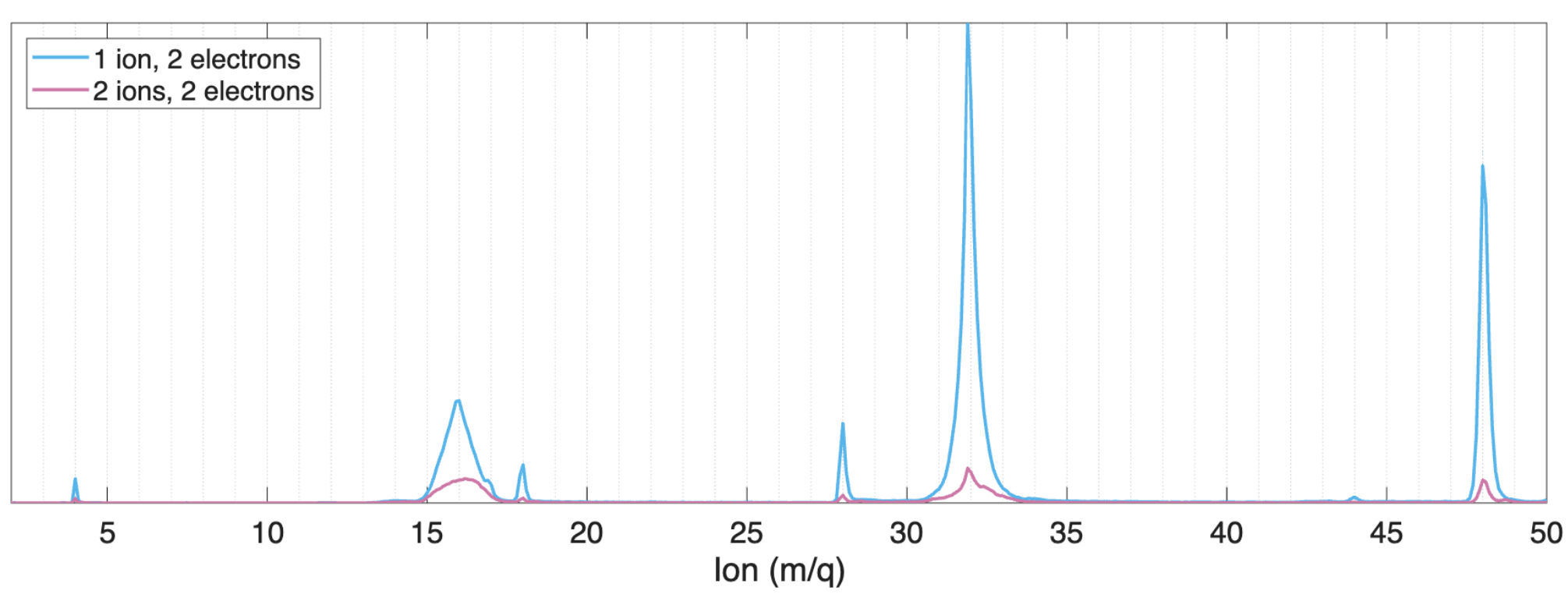

Figure S1. Mass spectrum of both single and two ions detected in coincidence with two electrons. Peaks at 16, (O^+), 32, (O_2^+) and 46 (O_3^+) stem from the sample. Signal from air is visible in the single-ion–two-electron spectrum at $m/q = 18$ (H_2O), 28 (N_2), and 44 (CO_2); the signal at $m/q = 4$ arises from He in the gas-discharge lamp. No trace of O_3^{2+} (which would appear at $m/q = 24$) is observed in either spectrum.

Table S1. Cartesian coordinate matrix obtained after geometry optimisation at the (R)CCSD(T)-F12b/aug-cc-pVTZ level of theory. Absolute energy in Hartree and harmonic frequencies with their respective symmetry are also given.

O_3 (X^1A_1)			-225.21594662	
	O	0.0000000000	-0.0000000000	0.4400339364
	O	0.0000000000	1.0810164025	-0.2200169682
	O	0.0000000000	-1.0810164025	-0.2200169682
		ν_1 (a_1)	ν_2 (a_1)	ν_3 (b_2)
		1175.53	727.92	1095.11
O_3^{2+} ($X^1\Sigma_g^+$)			-223.95164348	
	O	0.0000000000	-0.0000000000	0.0000654224
	O	0.0000000000	1.1676220132	-0.0000327112
	O	0.0000000000	-1.1676220132	-0.0000327112
		ν_1 (σ_g^+)	ν_2 (π_u)	ν_3 (σ_u^+)
		1056.73	417.29	1654.55
O_3^{2+} (A^3B_2)			-223.90957446	
	O	0.0000000000	0.0000000000	0.4172333388
	O	0.0000000000	1.1086297756	-0.2086166694
	O	0.0000000000	-1.1086297756	-0.2086166694
		ν_1 (a_1)	ν_2 (a_1)	ν_3 (b_2)
		982.40	559.40	586.47

Table S2. Adiabatic double ionization energies (ADIE in eV) of $O_3^{2+}(X^1\Sigma_g^+)$ and $O_3^{2+}(A^3B_2)$ calculated using the RCCSD(T)-F12/aug-cc-pVTZ (opt) // RCCSD(T)/ aug-cc-pVTZ + ΔCV + ΔSR + ΔZPVE (SP) composite scheme. The vertical double ionization energies (VDIE in eV) of $O_3^{2+}(X^1\Sigma_g^+)$ and $O_3^{2+}(A^3B_2)$ are also calculated following the same composite scheme excluding the ΔZPVE contributions.

	$O_3^{2+}(X^1\Sigma_g^+)$	$O_3^{2+}(A^3B_2)$	O_3 (X^1A_1)	$O_3^{2+}(X^1\Sigma_g^+)$	$O_3^{2+}(A^3B_2)$
PBE0/aug-cc-pVTZ (opt)	-223.9956634	-223.9741746	-225.2714113		
ZPE (PBE0/aug-cc-pVTZ) (eV)	0.226961179	0.136104972	0.208847538		
RCCSD(T)-F12/aug-cc-pVTZ (opt)	-223.9516435	-223.9095745	-225.2159466	-223.8990107	-223.9088958
RCCSD(T,F.C.)/cc-pwCVTZ (SP)	-223.8983826	-223.8577399	-225.1481896	-223.846658	-223.8566191
RCCSD(T,full)/cc-pwCVTZ (SP)	-224.0550069	-224.0138879	-225.3059049	-224.0014558	-224.0128177
RCCSD(T)/cc-pVTZ (SP)	-223.8836426	-223.8431194	-225.1326419	-223.832442	-223.8419878
RCCSD(T)/cc-pVTZ-DK (SP)	-223.8701241	-223.8295547	-225.1189072	-223.8189043	-223.8284305
	ADIE (eV)			VDIE (eV)	
RCCSD(T)-F12/aug-cc-pVTZ (opt)	34.403	35.548		35.836	35.567
ΔCV	0.029687365	0.042648427		0.079389532	0.041272075
ΔSR	-0.005883377	-0.004627571		-0.00536119	-0.004828391
ΔZPVE	0.018113641	-0.072742566			
RCCSD(T)-F12/aug-cc-pVTZ (opt) // RCCSD(T)/ aug-cc-pVTZ + ΔCV + ΔSR + ΔZPVE (SP)	34.445	35.513		35.910	35.603

Table S3. Calculated KERs as deduced from the MRCI/aug-cc-pV5Z 1D PES cuts of the ozone dication electronic states along the OO bond (Figure 5), toward the different considered asymptotes of the $O_2^+ + O^+$ fragmentation channels, from several starting points on the curves.

	Starting from the top of the barrier of			Starting from crossing between	
Channel	$X^1\Sigma_g^+$	A^3B_2	$3^1A''$	$X^1\Sigma_g^+$ - ($A^3B_2/1^3A''$)'	A^3B_2 - ($A^3B_2/1^3A''$)
O_2^+ ($X^2\Pi_g$) + O^+ (^4S)	11.62 [a)]	10.10 [b)]	12.78 [c)]	10.05 [d)]	9.70 [e)]
O_2^+ ($X^2\Pi_g$) + O^+ (^2D)	8.08 [a)]	6.55 [b)]	9.24 [c)]	6.50 [d)]	6.16 [e)]
O_2^+ ($X^4\Pi_u$) + O^+ (^4S)	5.47 [a)]	3.95 [b)]	6.63 [c)]	3.90 [d)]	3.56 [e)]
O_2^+ ($X^4\Pi_u$) + O^+ (^2D)	1.92 [a)]		3.08 [c)]		
O_2^{2+} ($X^1\Sigma_g^+$) + O (^3S)			2.31 [c)]		
O^+ (^4S) + O^+ (^4S) + O (^3P)	2.41 [f,g)]	2.30 [f,h)]	2.97 [f,i)]		

[a)] Appearance energy = 38.28 eV
[b)] Appearance energy = 36.76 eV
[c)] Appearance energy = 39.44 eV
[d)] Appearance energy = 36.71 eV
[e)] Appearance energy = 36.40 eV
[f)] CASSCF/aug-cc-pVTZ level
[g)] Appearance energy = 35.74 eV
[h)] Appearance energy = 35.63 eV
[i)] Appearance energy = 36.30 eV

Table S4. Spin-Orbit coupling matrix element between the $X^1\Sigma_g^+ - 1^3A''$ and $A^3B_2 - 1^3A''$ states at the positions of their respective crossings. The other OO distance and the in-plane OOO angle are set to 1.267 Å and 117.2° (cf. Figure 3). These spin-orbit integrals are evaluated in cartesian coordinates using the Breit–Pauli Hamiltonian as implemented in MOLPRO

	$R_{OO} = 1.403$ Å	$R_{OO} = 1.438$ Å
$\langle X^1\Sigma_g^+ \vert H_{SO} \vert 1^3A'' \rangle$	17.4i (x) / 7.4 (y)	-
$\langle A^3B_2 \vert H_{SO} \vert 1^3A'' \rangle$	-	22.8i(x) / 7.6 (y)

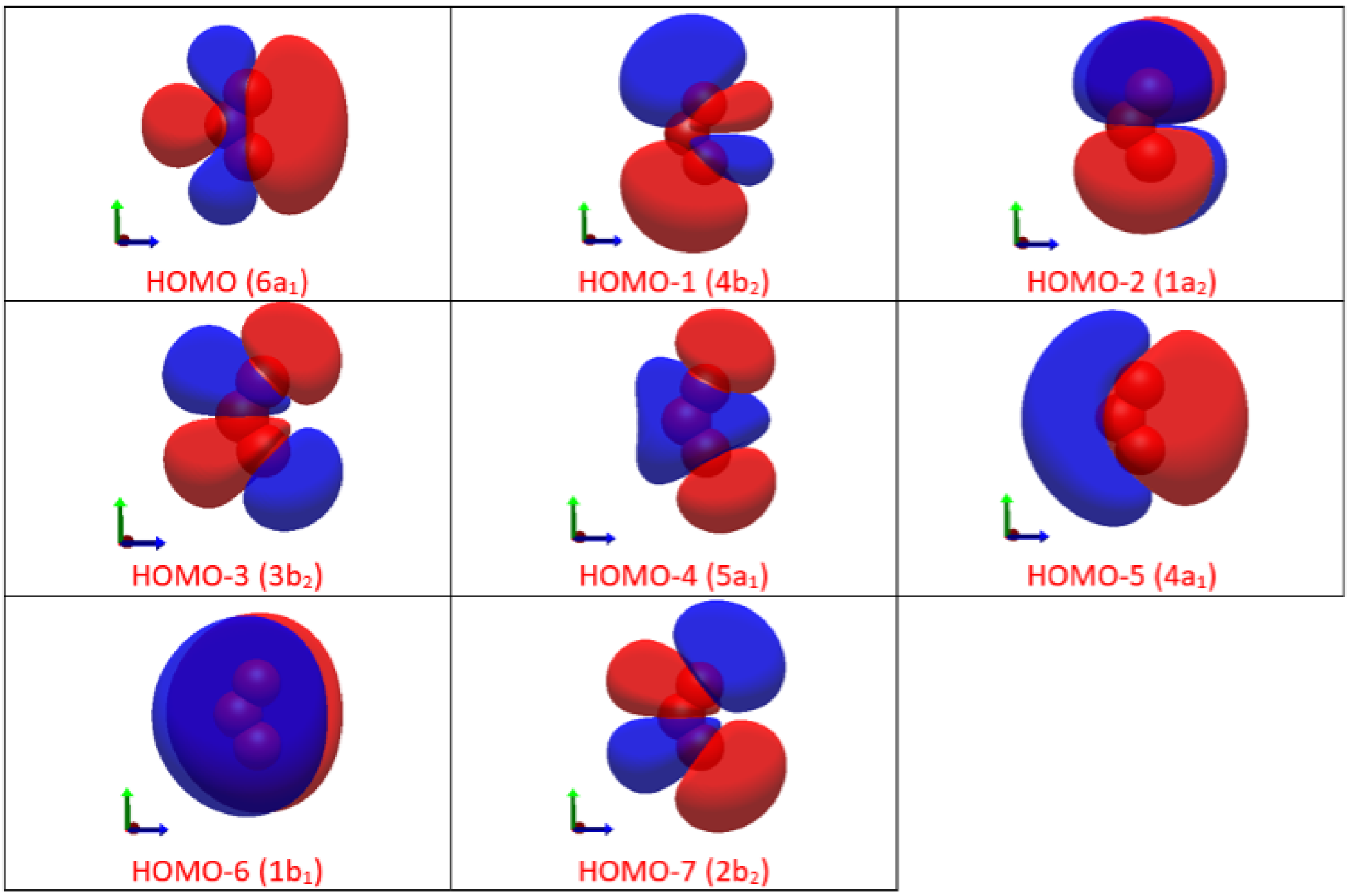

Figure S2. Visualization of the molecular orbitals of the neutral ozone molecule from HOMO to HOMO-7, as computed at the HF/aug-cc-pVTZ level of theory. The order and symmetry label of each orbital are given accordingly. Each column corresponds to a different convention for the choice of the C_2 symmetry axis of the C_{2v} point group, taken as the z axis. The small coordinate triads shown at the bottom-left of each orbital plot indicate the Cartesian axes used: x in red, y in green, and z in blue.
